# Failure analysis of a rapid quench boiler for ethylene cracker

**DOI:** 10.1016/j.heliyon.2024.e33961

**Published:** 2024-07-02

**Authors:** Zhihong Duan, Weiqi Lian, Pujie Zhan, Junde Song, Canyin Li, Xiangji Yang, Bingjiang Chen, Yunrong Lyu

**Affiliations:** a College of Mechanical and Electrical Engineering, Guangdong University of Petrochemical Technology, Maoming, 525000, China; b School of Energy and Power Engineering, Guangdong University of Petrochemical Technology, Maoming, 525000, China; c Guangdong Maohua Construction Group Co., Ltd, Maoming, 525000, China

**Keywords:** Rapid quench boiler, Corrosion leakage, High temperature oxygen corrosion, High temperature oxidation

## Abstract

The corrosion and leakage issues of rapid quench boilers have become increasingly prominent in ethylene plants, significantly disrupting the regular functioning of equipment. In pursuit of a more efficacious corrosion protection strategy, a study was conducted on the heat exchange tubes experiencing corrosion leakage in the quenching boiler of a petrochemical company. By means of macroscopic observation, chemical composition analysis, mechanical property analysis, metallographic analysis, fracture surface morphology observation, and energy spectrum analysis, combined with on-site process parameters, a comprehensive analysis of the failure causes of the corroded leakage sites was conducted. It was concluded that the perforation of the heat exchange tubes was caused by high-temperature oxygen corrosion and oxidation induced by scale accumulation, and reasonable countermeasures were proposed. According to X-ray diffraction analysis (XRD), it was found that the scale mainly consisted of Fe_2_O_3_ and Fe_3_O_4_, and the scale formation time was relatively long. It is speculated that the accumulation of scale is caused by the rust from the upstream equipment pipelines of the boiler water being carried into the quenching boiler with the fluid flow and accumulating at this location. Regarding the heat exchange tubes, the primary causes of failure are high-temperature oxygen corrosion and oxidation. To verify whether the relevant reactions can occur spontaneously, the critical transition temperature of the reactions is calculated using the free entropy function method. The calculated critical temperature for the occurrence of high-temperature oxygen corrosion and oxidation in the heat exchange tubes under failure conditions is determined to be T＜1058 K ~ 785 °C. Therefore, under the conditions of heat exchange tube failure, high-temperature oxygen corrosion and oxidation can occur spontaneously.

## 1 Introduction

The rapid quench boiler, as an essential reaction apparatus in ethylene cracking units, is primarily employed to rapidly cool the cracking gas produced by the cracking furnace [[1], [2], [3]]. Under the action of the rapid quench boiler, not only can the high-quality heat in the cracking gas be fully utilized to generate high-pressure steam for providing steam power to other equipment, but it can also enhance ethylene yield and prevent excessive coking. As one of the core components of ethylene cracking units, the rapid quench boiler plays a pivotal role in the production and construction of petrochemical enterprises [[4], [5], [6]]. There are numerous reasons for the failure of ethylene rapid quench boilers, such as material selection, variations in temperature fields, design strength of furnace tubes, defects in welding processes, uneven distribution of boiler feedwater, exceeding pH limits, coking and carburization. In addition, changes in working environments and operating durations may also lead to boiler failures [[7], [8], [9], [10], [11], [12]].

The corrosion issues resulting from the accumulation of various deposits and local high temperatures are particularly prominent in boiler corrosion research, with scale playing a significant role in many corrosion processes [13,14]. Husaini et al. [15]conducted a study on the problems of furnace cover leakage and outer tube rupture in waste heat boilers. They found that the improper geometric design of the furnace cover led to turbulence inside the cover, causing accelerated flow-induced corrosion. Local turbulence eroded the oxide layer at the bottom of the furnace cover, leading to the accumulation of oxides and deposits around the ring welds and closest fixed baffles inside the tubes, manifested as a thick layer of Fe_3_O_4_ deposits. The primary failure of the outer tubes of the waste heat boiler was attributed to inadequate water cooling, as leaks from the cover, combined with thick Fe_3_O_4_ oxide scale deposits on the fixed baffles, significantly raised the local temperature of the failed tubes, resulting in yielding deformation and lip rupture. Ren et al. [16]analyzed the causes of water-cooled wall tube leakage failure. Chemical composition analysis confirmed that the materials of the water-cooled wall tubes met technical requirements. Macroscopically, there was layering and peeling observed on the surface within the perforation area. Metallographic analysis revealed varying degrees of pearlite spheroidization, indicating localized overheating in the area. Energy spectrum analysis identified the accumulation of deposits as the primary factor causing corrosion. Combined macroscopic and microscopic analyses showed that the accumulation of deposits on the water-cooled wall tubes caused local overheating, concentration of corrosive media beneath the deposits, corrosion occurring beneath the deposits, and weakening of the metal material properties at high temperatures. When the wall thickness of the tubes decreased to a certain extent, it led to perforation leakage. Sun et al. [17]conducted a study on severe high-temperature corrosion problems occurring in a short period in 350 MW supercritical boiler water-cooled wall tubes. The high-temperature corrosion mechanism of the water-cooled wall tubes was analyzed through chemical analysis, metallographic examination, mechanical property testing, and scanning electron microscope observation. The results showed that high-temperature corrosion had no significant impact on material properties, and the main form of failure was deformation of the tube wall caused by corrosion. Structural and compositional analysis of the corrosion products revealed the presence of three layers of corrosion product layers on the outer surface of the water-cooled wall tubes. There were numerous pores between the corrosion product layers and the substrate, making the corrosion products prone to delamination. The sulfur content in the corrosion products increased gradually from the inside out, demonstrating that sulfur content was the primary factor influencing high-temperature corrosion of water-cooled wall tubes.

Boiler corrosion and leakage problems are not only prevalent in ethylene production units but are also commonly encountered in practical cases of other industrial productions [[18], [19], [20]]. Boilers, as important specialized equipment in the chemical and energy industries, often need to operate for long periods in harsh working environments characterized by high temperatures, high pressures, dust, and complex media. The risk of corrosion is high, and the causes of boiler corrosion leakage vary [[21], [22], [23]]. Failure to address these issues promptly can lead to losses for enterprises and even endanger public safety and property. Therefore, scientific and rational corrosion protection measures are essential.

This study presents a systematic study on high-temperature oxidation caused by scale accumulation, which results in poor heat transfer in heat exchange tubes due to design issues in rapid cooling boiler tube plates. Unlike common failures caused by erosion corrosion and corrosive media, this type of failure has received relatively little research attention and is often overlooked as a contributing factor. The research aims to fill this gap and provide a comprehensive understanding of the factors leading to high-temperature oxidation resulting from scale accumulation in heat exchange tubes.

These findings provide key technological support to ensure the long-term safe and stable operation of ethylene rapid quench boilers and enhance ethylene production capacity.

### 1.1 Summary of equipment failure and operating conditions

The rapid quench boiler of an ethylene cracking unit at a petrochemical company experienced a leakage after operating for just under 11 months. Relevant operational parameters during operation varied within reasonable ranges. However, during the inspection, it was discovered that four heat exchange tubes had developed perforation leaks approximately 300 mm from the inlet end of the cracking gas/boiler water. The design/operational parameters and materials of various components are presented in Table 1, Table 2.

**Table 1:** Processing parameter of quenching boiler.

	Tube side	Shell side
Design Pressure(MPaG)	0.45	13.31 + LH
Operating Pressure(MPaG)	0.08347	12.10 + LH
Design Temperature(°C)	Inlet:900/Outlet:525 heat exchange tube:385	350
Operating Temperature(°C)	Inlet:844/Outlet:345	324
Medium	Pyrolysis gas	water、steam

**Table 2:** Component materials of quenching boiler.

	Materials	Standard	Specification
Tube Sheet	12Cr2Mo1	NB/T47008-2017	Φ870 × 28
Heat exchange tube	13CrMo4-5	DIN EN10216-2	Φ63.5 × 7.1
Welding material	T Union GM Cr1Mo	AWS A5.28 NB/T47018	Φ1.2

## 2 Materials and methods

### 2.1 Materials and reagent

Component material of the failure quenching boiler is summarized as followed: The material and dimension of tube sheet is 12Cr2Mo1(meeting the standard of NB/T47008-2017) and Φ870 × 28 mm,respectively. The material and dimension of heat exchange tube is 13CrMo4-5(meeting the standard of DINEN10216-2) and Φ63.5 × 7.1 mm,respectively. The 4 % nitric acid ethanol solution is employed for metallographic testing. The solution is prepared by combining 45 % nitric acid solution (purity ≥45 %, Aladdin, Shanghai) with anhydrous ethanol (purity ≥99.5 %, anhydrous grade, moisture content ≤0.005 %, Aladdin, Shanghai).

### 2.2 Materials preparation

The material preparation in this study primarily involves the preparation of metallographic samples and the observation of samples obtained by direct cutting from failed components. The process of preparing metallographic samples includes sampling, embedding, grinding, polishing, and etching. Sample selection should be based on the research objectives, focusing on representative areas. Once the specific areas are identified, the samples can be sectioned. The dimensions of the samples typically consist of cylindrical samples with diameters of 12–15 mm and heights of 12–15 mm, or square samples with side lengths of 12–15 mm. The sampling method can involve the use of a handsaw, sawing machine cutting, or hammering. If the sample size is too small to be ground by hand, it can be embedded in a low-melting-point alloy or plastic to facilitate grinding and polishing. The cut or embedded samples are ground flat on a grinding machine, and sharp corners are rounded. Subsequently, coarse sandpaper is used for polishing, followed by a sequential fine grinding process using 400#, 600#, 800#, 1000#, 1200#, 1500#, 2000#, 2500#, and 3000# metallographic sandpaper, until a smooth finish is achieved. During the grinding process, the sample should be rotated 90° with each change of sandpaper. Additionally, finely ground samples require polishing to remove fine grinding marks and obtain a bright mirror surface. Polishing is carried out on a dedicated polishing machine at a speed of 100-150r/min. During polishing, the sample should be uniformly pressed against the rotating polishing disc and moved radially from the edge to the center. In addition to mechanical polishing, other methods such as electrolytic polishing and chemical polishing can also be employed. After polishing, the samples must undergo etching before observation under a microscope. Following etching, the samples are rinsed with water, then with alcohol, and finally dried with a blower before being placed on a metallographic microscope for observation.

### 2.3 Static and dynamic mechanical characterization

The chemical composition and mechanical performance testing in this study adheres to the following standards:

GB/T 222–2006 - Permissible deviations of chemical composition for finished steel products; GB/T 14203-2016 - General rules for spark discharge atomic emission spectrometric analysis; GB/T 13298-2015 - Methods for metallographic examination of metals; GB/T 17359-2012 - Quantitative analysis by microbeam analysis spectrometry; JB/T 6842-1993 - Test methods for scanning electron microscopy; GB/T30579-2014 - Damage mode recognition for pressure equipment;

NB/T47008-2017 - Forgings for pressure equipment made of carbon steel and alloy steel; DINEN10216-2 - Seamless steel tubes for pressure purposes - Technical delivery conditions Part 2: Alloy and non-alloy steel tubes with specified elevated temperature properties; GB/T3077 - Alloy structural steel.

These standards have been carefully selected to ensure the accuracy and reliability of the mechanical performance testing conducted in this study.

## 3 Results and discussion

### 3.1 Macroscopic observation

**Fig. 1:**
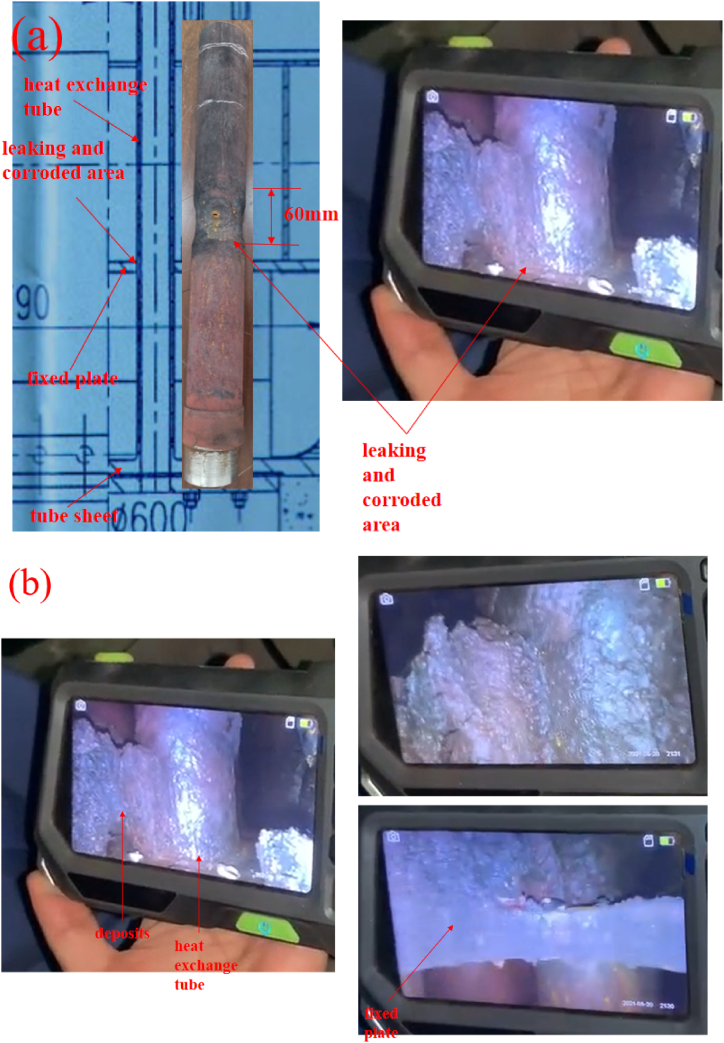
Macro observation of the failed pipe: (a)morphology of corroded and cracking area; (b)Endoscopy images of deposit accumulation and corroded area.

As depicted in Fig. 1a-b, the leakage site of the rapid quench boiler is located above the fixed plate at the inlet end of the cracking gas/boiler water. The corrosion is highly localized, occurring only within approximately 60 mm above the fixed plate. Within this range, significant corrosion thinning is observed along the entire circumference of the heat exchange tubes, while no corrosion thinning is evident in other areas of the tubes. On-site endoscopic inspection reveals severe fouling at the corroded thinning site (above the fixed plate), with the corroded areas precisely corresponding to the fouled regions. Combining the above analysis, the spatial arrangement formed between the root of the fixed plate at the inlet end of the cracking gas/boiler water and the junction of the heat exchange tubes facilitates the accumulation of residual boiler water and fouling, which is highly correlated with the occurrence of corrosion thinning phenomena.

**Fig. 2:**
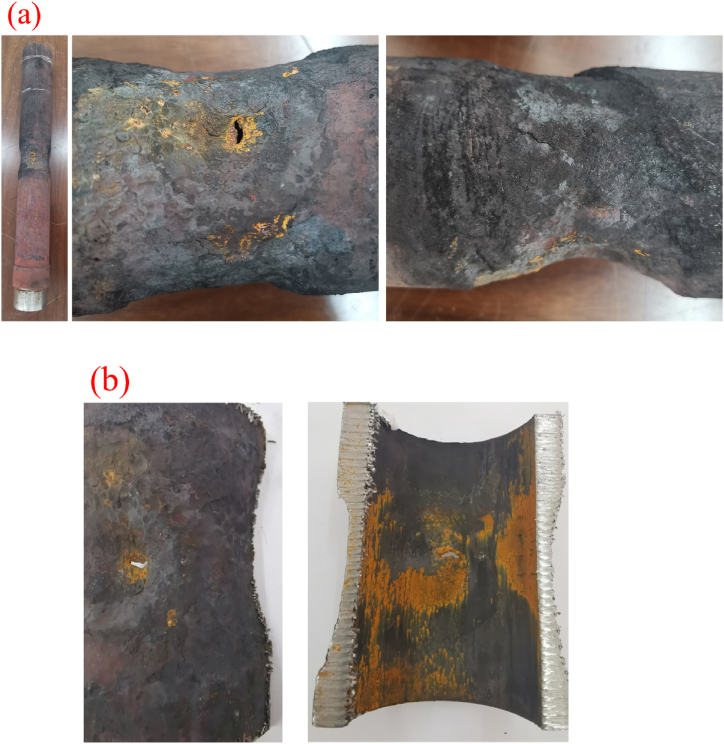
Morphology of failure heat exchange tube:(a) outer wall; (b) inner wall.

Fig. 2a-b illustrates the macroscopic morphology of corroded heat exchange tubes. From the figure, it can be observed that corrosion primarily occurs on the outer wall (boiler water side), while the inner wall (cracking gas side) exhibits no significant corrosion. This indicates that the heat exchange tubes primarily experience external corrosion. When the corrosion thins the wall to a point where it can no longer withstand external pressure (approximately 12 MPa), the tubes undergo a collapse instability deformation from the outer wall to the inner wall, eventually leading to rupture and leakage. The length of the rupture leakage point is approximately 8 mm, with a width of about 2 mm.

The corroded and thinned sections of the heat exchange tubes are covered with black and yellow-brown deposits, some of which exhibit a laminar distribution. Upon removal of these deposits, the surface reveals densely distributed pits with relatively smooth bottoms.

Based on the observed corrosion morphology and deposit accumulation, it is inferred that localized corrosion of the heat exchange tubes is attributed to high-temperature oxygen corrosion and oxidation in the boiler water. Due to design issues related to rapid cooling boilers, deposits tend to accumulate at the root of the tube fixed plate and the junction of the heat exchange tube, affecting heat transfer. This leads to an increase in the wall temperature of the heat exchange tubes, resulting in high-temperature oxygen corrosion and oxidation at the sites of deposit accumulation (the pits are attributed to high-temperature oxygen corrosion, while the laminar deposits are attributed to oxidation).

### 3.2 Chemical and mechanical properties analysis

Chemical composition analysis was conducted on the sampled specimens, and the results are presented in Table 3. The analysis indicates that the chemical composition of the heat exchange tube aligns with the requirements specified for 13CrMo4-5 material in the DIN EN 10216-2 standard.

**Table 3:** Chemical composition analysis.

Sample	Element content(%)							
	C	Si	Mn	P	S	Cr	Mo	Ni
Heat exchange tube	0.14	0.23	0.470	0.019	0.007	1.04	0.548	/
13CrMo4-5	0.10–0.17	≤0.35	0.40–0.70	≤0.025	≤0.020	0.70–1.15	0.40–0.60	≤0.30
15CrMo	0.12–0.18	0.17–0.37	0.40–0.70	≤0.035	≤0.035	0.80–1.1.0	0.40–0.55	≤0.30

Mechanical performance and hardness testing were conducted on the sampled specimens, and the results are presented in Table 4, Table 5, Table 6. The analysis indicates that the heat exchange tube meets the standard requirements for yield strength, tensile strength, elongation at fracture, impact toughness, and hardness.

**Table 4:** Results of mechanical properties.

	Yield strength(MPa)	Tensile strength(MPa)	Fracture elongation(%)
Heat exchange tube base	377	479	35.7
Leaking heat exchange tube	383	492	32.2
13CrMo4-5 DINEN10216-2	≥290	440–590	≥22

**Table 5:** Results of impact test.

	Impact energy(Akv/J)	Average(Akv/J)	
Heat exchange tube	128/134/130	131	Small sample(5 × 10 × 55)
13CrMo4-5 DINEN10216-2	40

**Table 6:** Results of hardness.

	1	2	3	4	5
Heat exchange tube	141	142	152	149	149
12Cr2Mo1 NB/T47008-2017	125–180				

### 3.3 Boiler water quality analysis

**Fig. 3:**
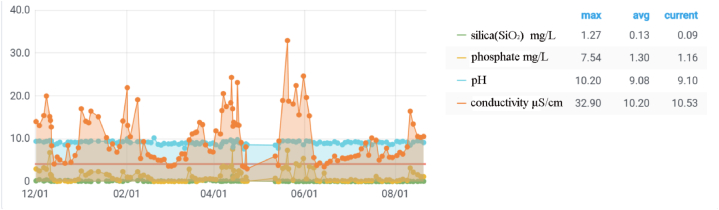
Analysis of boiler water quality.

To identify the source of deposits accumulated at the root of the fixed plate of the heat exchange tube and the junction with the heat exchange tube, sampling data on the boiler water quality from the enterprise were retrieved for analysis. The data covers the period nine months prior to the failure of the heat exchange tube. The analysis results are as follows:

According to the national standard GB/T 12145, the requirements for boiler water quality under an internal steam drum pressure of 12.7–15.6 MPa are as follows: conductivity <20 μS/cm; silica (SiO_2_) ≤0.45 mg/L; phosphate ≤3 mg/L; pH value: standard range: 9.0–9.7; target range: 9.3–9.7. As shown in Fig. 3, the enterprise conducted sampling analysis for the pH value, conductivity, silica content, and phosphate content of the boiler water. Although some data fluctuated significantly, the average values of water quality parameters over nine months generally fell within the range of the national standard. The average data for water quality over nine months are as follows: conductivity 10.53 μS/cm; silica (SiO_2_) 0.09 mg/L; phosphate 1.16 mg/L; pH value: 9.1.

In summary, the boiler water quality largely meets the requirements of the national standard, thereby excluding the influence of boiler water on corrosion thinning.

### 3.4 Metallographic analysis

**Fig. 4:**
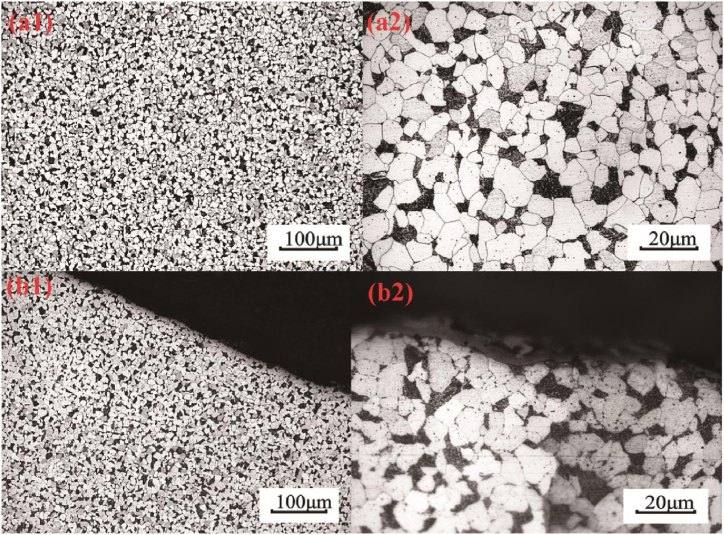
Metallographical structure of the (a1-a2) matrix and (b1-b2) the leaking part.

Samples were taken from the heat exchange tube at locations distant from the corrosion and corrosion pit sites for optical metallographic observation. Fig. 4a1-4a2 shows the metallographic structure of the base material of the heat exchange tube away from the corroded leakage site, exhibiting a pearlite + ferrite structure with slight spheroidization of localized pearlite. Fig. 4b1-4b2 depicts the metallographic structure of the corroded area of the heat exchange tube, presenting a pearlite + ferrite structure. However, the pearlite has undergone moderate spheroidization, and carbides precipitate along the grain boundaries in blocky and chain-like forms, indicating prolonged exposure to high temperatures at this corroded site.

### 3.5 Fracture surface scanning electron microscopy analysis

**Fig. 5:**
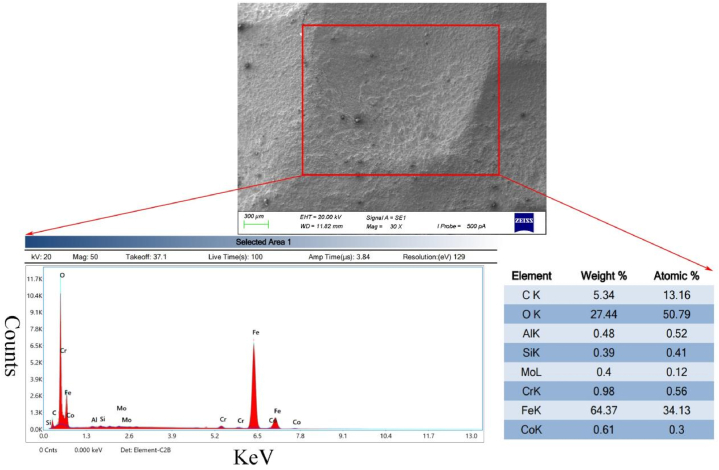
Morphology and Energy dispersive spectrum analysis of corrosion pits.

To further analyze the process and characteristics of corrosion, scanning electron microscopy was employed to investigate the morphology and elemental composition of the corrosion pits. Fig. 5 presents the corrosion morphology of the pits along with the results of energy-dispersive X-ray spectroscopy (EDS) analysis of the corrosion products. It can be observed that the surface of the pits appears relatively smooth and flat at low magnification, with localized flaky deposits, while at higher magnifications, porous corrosion products are evident. EDS analysis reveals that the corrosion products on the pit surface consist of metals and metal oxides, predominantly iron oxides, further indicating that the corrosion of the heat exchange tube is attributed to high-temperature oxygen corrosion and oxidation in the boiler water.

### 3.6 Analysis of deposits: energy-dispersive X-ray spectroscopy and X-ray diffraction

**Fig. 6:**
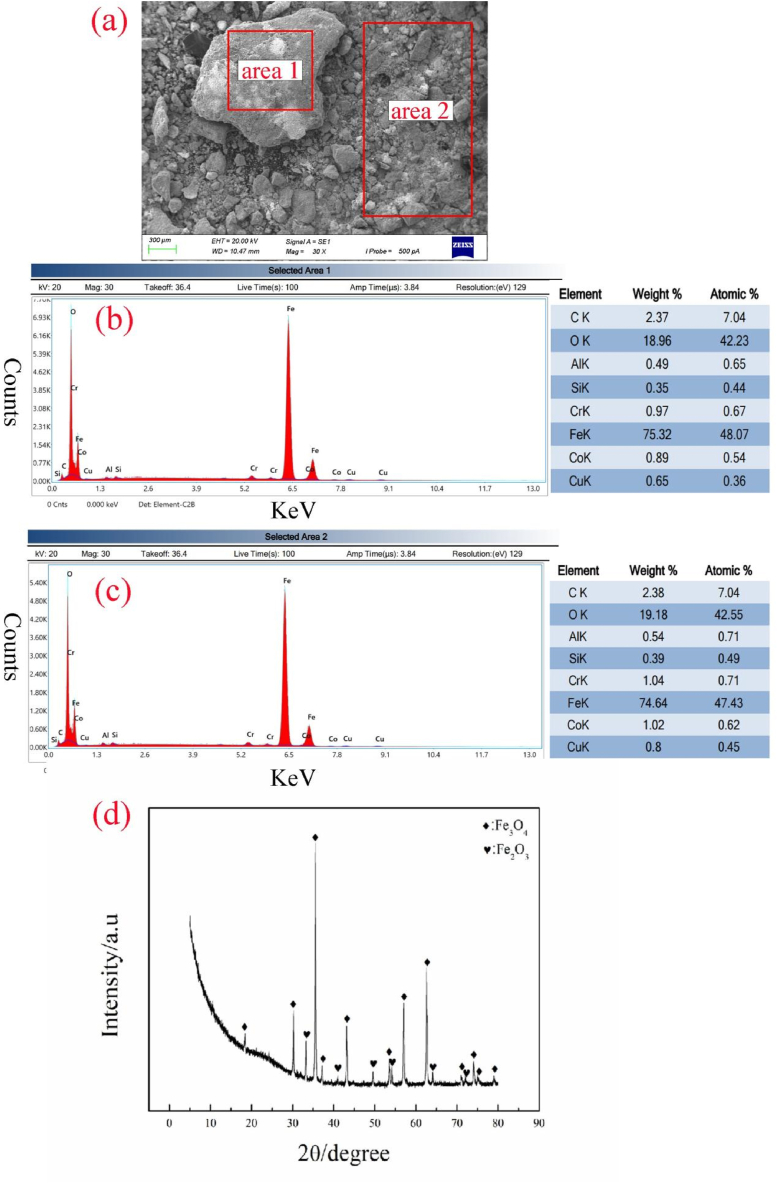
Energy dispersive spectrum and XRD analysis of fouling product (a) EDS scanning area; (b) result of area 1; (c) result of area 2; (d) XRD results of fouling product.

Sampling of deposits on the surface of the corroded section of the heat exchange tube was conducted for energy-dispersive X-ray spectroscopy analysis, with the results presented in Fig. 6a-d. The analysis reveals that the composition of the deposits on the surface of the corroded section of the heat exchange tube is consistent with the corrosion products near the pit surface, consisting of metals and metal oxides, predominantly iron oxides. X-ray diffraction analysis of the deposits, as shown in Fig. 6d, indicates that the deposits are primarily composed of Fe_2_O_3_ (PDF#89–8103,Hematite,γ-Fe_2_O_3_) and Fe_3_O_4_ (PDF#75–0449,Magnetite).

### 3.7 Thermodynamic analysis of corrosion mechanisms

Based on the comprehensive analysis above, it can be inferred that the localized corrosion and thinning of the heat exchange tube are attributed to high-temperature oxygen corrosion and oxidation in the boiler water. The deposits initially accumulate at the root of the heat exchange tube's fixed plate, impeding heat transfer, thereby leading to an increase in the local wall temperature of the heat exchange tube. Consequently, rapid high-temperature oxygen corrosion and oxidation occur at the site of deposit accumulation (high-temperature oxidation refers to the process where metals react with oxygen at elevated temperatures to form metal oxides; dissolution corrosion of metals in boiler water is an electrochemical corrosion, and both types of corrosion accelerate with increasing temperature). When the corrosion thinning exceeds the wall thickness's ability to withstand external pressure (approximately 12 MPa), the heat exchange tube undergoes collapse deformation from the outer wall to the inner wall, ultimately rupturing and causing leakage. Thus, the primary cause of corrosion perforation and leakage in the heat exchange tube is the accumulation of deposits at the root of the heat exchange tube's fixed plate.

Whereas Fe(OH)_2_ is unstable and prone to further reaction, ultimately forming Fe_3_O_4_. As depicted in the above reaction equations, as long as dissolved oxygen exists in the boiler water, metal oxidation corrosion occurs, and the corrosion rate accelerates with increasing oxygen content and water temperature. Oxygen corrosion typically manifests as localized corrosion with ulcers and pinholes, covered by yellow-brown or black deposits. High-temperature oxidation refers to the process where metals react with oxygen at high temperatures to form metal oxides, accelerating with increasing temperature.

To verify whether the aforementioned reactions can spontaneously occur, the critical transition temperatures of the reactions were calculated using the Gibbs free energy function. Corrosion of metals in boiler water by dissolved oxygen is an electrochemical corrosion process where iron and oxygen form two electrodes, constituting a corrosion cell. Iron always has a lower electrode potential than oxygen; in the iron-oxygen corrosion cell, iron acts as the anode and undergoes corrosion, while oxygen serves as the depolarizer and undergoes reduction, as described by the following reaction equation:

(3-1) $$\text{Anode}:Fe\left(s\right)\to Fe^{2+}\left(aq\right)+2e^{-}$$

Firstly, the standard entropy and standard enthalpy values of reaction equation 3-1 at a temperature of 298 K were determined based on thermodynamic the standard entropy change: $\Delta_{r}S_{m,T}^{\theta }$ (Fe^2+^) = 87.8 J/mol·K; $\Delta_{r}H_{m,T}^{\theta }$ (Fe^2+^) = 0 kJ/mol; $\Delta_{r}S_{m,T}^{\theta }$ (Fe) = 27.33 J/mol·K; $\Delta_{r}H_{m,T}^{\theta }$ (Fe) = 0 kJ/mol

According to fundamental thermodynamic principles

(3-2) ΔG=ΔH−TΔS

(3-3) $$\Delta H=\Delta H_{\text{products}}-\Delta H_{\text{reactants}}$$

(3–4) $$\Delta S=\Delta S_{\text{products}}-\Delta S_{\text{reactants}}$$

substituting the above data to equation 3-2, 3-3 and 3–4, the results of entropy change and enthalpy change can be calculated as followed:

ΔH = 0 kJ/mol

ΔS = 60.47 J/(mol·K)

Hence, at 298 K, the entropy change of the reaction $Fe\left(s\right)\to Fe^{2+}\left(aq\right)+2e^{-}$ is 60.47 J/mol·K, and the enthalpy change is 0 kJ/mol. At this temperature, ΔH = 0, and ΔS>0. In this scenario, according to fundamental thermodynamic principles ΔG=ΔH−TΔS, at 298 K, ΔG must be less than 0. However, the operating temperature of the heat exchange tubes is 324 °C, approximately 597 K, necessitating the calculation of the reaction equilibrium critical temperature. Upon substitution of the aforementioned values, ΔG remains 0. Consequently, the reaction can proceed spontaneously at the operating temperature of the heat exchange tubes.

At that time, when ΔG＜0, ΔH＜TΔS,upon substitution of the above values, the result obtained was T＞0. Therefore, the reaction can proceed spontaneously at the operating temperature of the heat exchange tubes.

(3–5) $$\text{Cathode}:O_{2}\left(g\right)+2H_{2}O\left(l\right)+4e^{-}\to 4OH^{-}$$

According to thermodynamic data, the standard molar entropies at 298 K for reaction equation 3-5 are as follows: The standard entropy of O_2_ $\Delta_{r}S_{m,T}^{\theta }$ (O_2_) is 160 J/(mol·K); the standard enthalpy of O_2_ $\Delta_{r}H_{m,T}^{\theta }$ (O_2_) is 0 kJ/mol; the standard entropy of H_2_O $\Delta_{r}S_{m,T}^{\theta }$ (H_2_O) is 70 J/(mol·K); the standard enthalpy of H_2_O $\Delta_{r}H_{m,T}^{\theta }$ (H_2_O) is −285.8 kJ/mol; the standard entropy of OH^−^ $\Delta_{r}S_{m,T}^{\theta }$ (OH^−^) is 40 J/(mol·K); and the standard enthalpy of OH^−^ $\Delta_{r}H_{m,T}^{\theta }$ (OH^−^) is −230 kJ/mol.

By substituting the above values into equation 3-2, 3-3 and 3–4, the calculation can be performed to obtain the result as followed:

ΔS = −140 J/(mol·K)

ΔH = −348.4 kJ/mol

Therefore, at 298 K, the standard entropy change of the reaction $O_{2}\left(g\right)+2H_{2}O\left(l\right)+4e^{-}\to 4OH^{-}$ is −140 J/(mol·K), the standard enthalpy change is −348.4 kJ/mol, and at 298 K, ΔG < 0. However, the operating parameters of the heat exchange tube are 324 °C ~ 597 K, necessitating the calculation of the critical temperature range for reaction equilibrium. When ΔH < 0 and ΔS < 0, under these conditions, to ensure △G < 0, it is necessary to minimize the absolute value as much as possible. Substituting the aforementioned values, the critical temperature is calculated to be 2488.57 K ~ 2215 °C. Therefore, the reaction can proceed spontaneously at the operating temperature of the heat exchange tube.

The corrosion resulting from the above reaction is oxygen depolarization corrosion, wherein iron undergoes dissolution oxygen corrosion to produce Fe^2+^. The secondary reaction occurring in water is:

(3–6) $$Fe^{2+}\left(aq\right)+2OH^{-}\to Fe{\left(OH\right)}_{2}$$

(3–7) $$Fe{\left(OH\right)}_{2}\left(s\right)+2H_{2}O+O_{2}\to 4Fe{\left(OH\right)}_{3}\left(s\right)$$

(3–8) $$Fe{\left(OH\right)}_{2}\left(s\right)+2Fe{\left(OH\right)}_{3}\left(s\right)\to Fe_{3}O_{4}+4H_{2}O$$

According to thermodynamic data, the following information relevant to reaction equation 3-6 can be obtained: $\Delta_{r}S_{m,T}^{\theta }$ (Fe^2+^) = 77.6 J/mol·K; $\Delta_{r}S_{m,T}^{\theta }$ (OH^−^) = −10.8 J/mol·K; $\Delta_{r}S_{m,T}^{\theta }$ (Fe(OH)_2_) = 20.2 J/mol·K; $\Delta_{r}H_{m,T}^{\theta }$ (Fe^2+^) = 0 kJ/mol; $\Delta_{r}H_{m,T}^{\theta }$ (OH^−^) = −229.6 kJ/mol; $\Delta_{r}H_{m,T}^{\theta }$ (Fe(OH)_2_) = −561.2 kJ/mol

By substituting the above values into equation 3-2, 3-3 and 3–4, the calculation can be performed to obtain the result as followed:

ΔS = −96.4 J/mol·K

ΔH = −102.0 kJ/mol

Therefore, the standard enthalpy change for reaction equation 3-6 at 298 K is −102.0 kJ/mol. At this temperature, both ΔH and ΔS are negative, indicating spontaneity of the reaction at 298 K. However, the operational temperature of the heat exchange tube is 324 °C, approximately 597 K. It is necessary to calculate the critical temperature range for reaction equilibrium. In cases where ΔH is negative and ΔS is negative, to drive ΔG to be negative, one must minimize the absolute values. Substituting the above values, the critical temperature is found to be 1058 K, approximately 785 °C. Therefore, the reaction equation 3-6 can occur spontaneously at the operating temperature of the heat exchange tube.

According to thermodynamic data, the following information relevant to reaction equation 3-7 can be obtained:For Fe(OH)_2_: $\Delta_{r}S_{m,T}^{\theta }$ = 80 J/(mol·K) $\Delta_{r}H_{m,T}^{\theta }$ = −250 kJ/mol; for H_2_O: $\Delta_{r}S_{m,T}^{\theta }$ = 70 J/(mol·K); $\Delta_{r}H_{m,T}^{\theta }$ = −285.8 kJ/mol; for Fe(OH)_3_: $\Delta_{r}S_{m,T}^{\theta }$ = 90 J/(mol·K); $\Delta_{r}H_{m,T}^{\theta }$ = −500 kJ/mol.

By substituting the above values into equation 3-2, 3-3 and 3–4, the calculation can be performed to obtain the result as followed:

ΔS = −160 J/(mol·K)

ΔH = −428.4 kJ/mol

Therefore, the standard entropy of the reaction equation 3-7 is −160 J/(mol·K), and the standard enthalpy is −428.4 kJ/mol. At this point, both ΔH and ΔS are less than 0, indicating through calculations that the reaction can spontaneously occur at 298 K. However, the operational parameters of the heat exchange tubes are 324 °C, approximately 597 K, necessitating the determination of the critical temperature range for reaction equilibrium. Given ΔH < 0 and ΔS < 0, to achieve ΔG < 0, it is imperative to minimize their absolute values. By substituting the aforementioned numerical values, a critical temperature of 2677.5 K, approximately 2404.5 °C, is obtained. Hence, the reaction equation 3-7 can spontaneously occur at the operating temperature of the heat exchange tubes.

According to thermodynamic data, the following information relevant to reaction equation 3-8 can be obtained:for Fe(OH)_2_: $\Delta_{r}S_{m,T}^{\theta }$ = 80 J/(mol·K); $\Delta_{r}H_{m,T}^{\theta }$ = −250 kJ/mol; for Fe(OH)_3_: $\Delta_{r}S_{m,T}^{\theta }$ = 90 J/(mol·K); $\Delta_{r}H_{m,T}^{\theta }$ = −500 kJ/mol; for Fe_3_O_4_: $\Delta_{r}S_{m,T}^{\theta }$ = 100 J/(mol·K); $\Delta_{r}H_{m,T}^{\theta }$ = −600 kJ/mol; for H_2_O: $\Delta_{r}S_{m,T}^{\theta }$ = 70 J/(mol·K); $\Delta_{r}H_{m,T}^{\theta }$ = −285.8 kJ/mol.

By substituting the above values into equation 3-2, 3-3 and 3–4, the calculation can be performed to obtain the result as followed:

ΔS = −90 J/(mol·K)

ΔH = −493.2 kJ/mol

Therefore, the standard entropy of reaction equation 3-8 is −90 J/(mol·K), and the standard enthalpy is −493.2 kJ/mol. At this juncture, both ΔH and ΔS are less than 0, indicating through calculations that the reaction can spontaneously occur at 298 K. However, the operational parameters of the heat exchange tubes are 324 °C, approximately 597 K, necessitating the determination of the critical temperature range for reaction equilibrium. Given ΔH ＜0 and ΔS ＜0, in such circumstances, to ensure ΔG ＜0, it is imperative to minimize their absolute values. Substituting the aforementioned numerical values, a critical temperature of 5480 K, approximately 5207 °C, is obtained. Hence, the reaction equation 3-8 can spontaneously occur at the operating temperature of the heat exchange tubes.

In conclusion, under the operational conditions of the heat exchange tubes, the aforementioned reactions can all proceed spontaneously, demonstrating that the failure mechanism at the corrosion site of the heat exchange tube is attributed to high-temperature oxidation and oxygen corrosion in boiler water.

### 3.8 Discussion

Macroscopic observations indicate that the leakage site of the quench cooler in a petrochemical enterprise is located above the fixed plate at the inlet of the cracking gas/boiler water. The corrosion is extremely localized, occurring only within a range of approximately 60 mm above the fixed plate. Within this range, there is significant corrosion thinning along the entire circumference of the heat exchange tube, while no corrosion thinning is observed at other parts of the heat exchange tube. Corrosion primarily occurs on the outer wall (boiler water side), with the corroded surface adhering to black and yellow-brown deposits. Some of the deposits exhibit laminar distribution, and after their removal, the surface is densely pitted, with relatively smooth pit bottoms. On-site endoscopic inspection reveals severe fouling at the corroded and thinned section of the heat exchange tube (above the fixed plate), corresponding precisely to the location of fouling.

Based on the aforementioned corrosion morphology and fouling accumulation, it is inferred that the localized corrosion of the heat exchange tube is caused by high-temperature oxygen corrosion and oxidation of the boiler water. The accumulation of fouling at the base of the fixed plate of the heat exchange tube affects heat transfer, resulting in an increase in the local wall temperature of the heat exchange tube. This leads to the occurrence of high-temperature oxygen corrosion and oxidation at the fouling accumulation site (pitting corrosion is caused by high-temperature oxygen corrosion, while laminar deposits are caused by oxidation). When the corrosion thinning reaches a point where the wall thickness can no longer withstand external pressure (approximately 12 MPa), internal concave instability deformation occurs, leading to leakage.

Chemical composition analysis results indicate that the chemical composition of the heat exchange tube complies with the requirements of the DIN EN 10216-2 standard for the 13CrMo4-5 material.

Mechanical property and hardness testing demonstrate that the yield strength, tensile strength, elongation at break, impact toughness, and hardness of the heat exchange tube all meet the standard requirements.

Metallographic observation results show that the parent metal microstructure of the heat exchange tube, away from the corroded area, consists of pearlite + ferrite structure with a grain size of 9–10 according to DIN EN 10216-2, corresponding to the normalized state of 13CrMo4-5 steel (equivalent to Chinese 15CrMo). There is slight spheroidization of local pearlite. The parent metal microstructure of the corroded area of the heat exchange tube consists of pearlite + ferrite structure, with moderate spheroidization of pearlite observed. Additionally, carbides are observed to precipitate along the grain boundaries in blocky and chain-like forms, indicating prolonged exposure to high temperatures at the corroded site, with the formation of fouling over an extended period.

Electron microscopy scanning and energy spectrum analysis reveal that the pit surface appears relatively smooth and flat at low magnifications, with locally present layered deposits. At higher magnifications, the corrosion products appear porous. Energy spectrum and XRD analysis indicate that the corrosion products on the pit surface consist of metal and metal oxides, predominantly composed of Fe oxides (Fe_2_O_3_ and Fe_3_O_4_), further confirming that the corrosion of the heat exchange tube is due to high-temperature oxygen corrosion and oxidation of the boiler water.

Fouling energy spectrum and XRD analysis demonstrate similar compositions of fouling at various sites, consisting mainly of metal and metal oxides, primarily Fe oxides (Fe_2_O_3_ and Fe_3_O_4_).

Comprehensive thermodynamic analysis results demonstrate that high-temperature oxygen corrosion and oxidation of the boiler water can spontaneously occur in service environments, further confirming that the dominant mechanism leading to the failure of the heat exchange tube is corrosion thinning caused by high-temperature oxygen corrosion and oxidation of the boiler water, eventually leading to instability and rupture leakage.

## 4 Conclusion

1. The chemical composition and mechanical properties of the leaking heat exchange tube in the ethylene cracking unit of a petrochemical enterprise comply with the requirements of the DIN EN 10216-2 standard for the 13CrMo4-5 material.
2. The corrosion thinning of the leaking heat exchange tube in the quench cooler of the ethylene cracking unit of a petrochemical enterprise is attributed to high-temperature oxygen corrosion and oxidation of the boiler water. Fouling initially accumulates at the base of the fixed plate of the heat exchange tube, affecting heat transfer and causing an increase in the wall temperature of the heat exchange tube. This leads to accelerated high-temperature oxygen corrosion and oxidation at the fouling accumulation site. When the corrosion thinning reaches a point where the wall thickness can no longer withstand external pressure (approximately 12 MPa), the heat exchange tube undergoes concave instability deformation from the outer wall towards the inner wall, ultimately rupturing and causing leakage.
3. Based on the fact that the fouling is mainly composed of Fe_2_O_3_ and Fe_3_O_4_ and has accumulated over a long period (confirmed through metallographic observations that the parent metal pearlite structure at the corroded section of the heat exchange tube has undergone moderate spheroidization, with carbides precipitating along the grain boundaries in blocky and chain-like forms, indicating prolonged exposure to high temperatures, further validating the long formation period of the fouling), it is speculated that the accumulation of fouling is due to the rust from the upstream equipment pipelines of the boiler water being carried into the quench cooler by the fluid flow and accumulating at that location.

## 5 Countermeasures

1. Examination of similar quench coolers in boilers to determine if there is a similar fouling phenomenon at the junction of the fixed plate and the heat exchange tube. If fouling is present, it should be promptly cleaned, and the extent of corrosion thinning of the heat exchange tube should be inspected.
2. Further analysis to confirm the source of the fouling and implement improvements to prevent a recurrence of similar incidents.

## Funding

Financial support by the Projects of Talents Recruitment of 10.13039/100008963Guangdong University of Petrochemical Technology (No.2022RCYJ2005), Maoming Science and Technology Plan Project(No.2023019) and the Open Foundation of Guangdong Provincial key laboratory of Petrochemical Equipment Fault Diagnosis (No.91720205) are gratefully acknowledged.

## CRediT authorship contribution statement

**Zhihong Duan:** Supervision, Investigation, Formal analysis, Data curation, Conceptualization. **Weiqi Lian:** Writing – review & editing, Writing – original draft, Supervision, Investigation, Funding acquisition, Formal analysis, Data curation, Conceptualization. **Pujie Zhan:** Writing – review & editing, Writing – original draft, Funding acquisition, Data curation, Conceptualization. **Junde Song:** Methodology, Investigation. **Canyin Li:** Methodology, Formal analysis. **Xiangji Yang:** Methodology, Investigation. **Bingjiang Chen:** Methodology, Investigation. **Yunrong Lyu:** Methodology, Investigation, Data curation, Conceptualization.

## Declaration of competing interest

The authors declare that they have no known competing financial interests or personal relationships that could have appeared to influence the work reported in this paper.
